# Is Heart Failure a New Risk Factor for Incident Cancer?

**DOI:** 10.3389/fcvm.2022.828290

**Published:** 2022-02-07

**Authors:** Xueyang Zheng, Na Li, Yanda Zhang, Jian Zhao

**Affiliations:** ^1^Institute of Organ Transplantation of PLA, Second Affiliated Hospital of Naval Medical University, Shanghai, China; ^2^Department of Cardiology, Second Affiliated Hospital of Naval Medical University, Shanghai, China

**Keywords:** heart failure, cancer, epidemiology descriptive, meta-analysis, risk factors

## Introduction

Cancer and heart failure (HF) are two leading global causes of morbidity and mortality and bring a huge disease burden to the world. Although cancer and HF commonly coexist, HF is often considered as a complication secondary to the cardio-toxicity of anti-cancer treatments and much attention has been paid to HF incidence among cancer patients. On the contrary, cancer development as a consequence of HF, has been far less studied. However, in recent years, accumulating evidence has suggested HF and cancer share lots of common risk factors and have various similarities. Inflammation, obesity, oxidative stress, diabetes, hypertension, and others are all contributors to the development of HF and cancer (1, 2). It seems that there is a bidirectional relationship between cancer and HF, the co-occurrence of which may be promoted by a common pathological milieu characterized by a state of chronic low-grade inflammation (3).

In view of this, it is plausible to presume that patients with HF are more prone to developing incident cancer. Several epidemiological studies have been done to identify whether patients with HF have an increased risk of incident cancer, but results from these studies are inconsistent or even conflicting. On one side, some previous studies reported a higher risk of malignancy in subjects with HF than in non-HF controls (4, 5); on the other side, some studies just revealed HF was not associated with an increased risk of cancer (6, 7)_._ Although a previous meta-analysis of only 4 studies demonstrated a correlation between HF and increased cancer risk (8), more large prospective cohort studies have been published in recent years and showed some difference (4, 5, 7, 9). In order to further investigate the association between HF and the risk of incident cancer, we conducted an updated meta-analysis of published cohort studies.

## Methods and Findings

Two investigators independently reviewed published studies in PubMed, EMBASE and Web of Science databases from their inception to October 2021 using the search strategy that included the terms for “HF” and “incident cancer.” Then, we manually searched for additional studies using references of selected retrieved articles to identify other possible studies. There was no limitation on language. Studies were included according to the following inclusion criteria: 1. cohort study; 2. HF was diagnosed before cancer incidence; 3. hazard ratio (HR) or equivalents with 95% confidential interval (CI) was reported. We excluded experimental studies, clinical trials, cross-sectional studies, case–control studies, reviews, commentaries, letters and conference abstracts. Data extraction was completed independently by two investigators, using a predefined data extract form. Disagreements were resolved by discussion among all investigators. Information on study characteristics, participants' features, exposure and outcomes were extracted. If both the crude and adjusted values were provided, we only extracted the adjusted values. The analysis was performed using Stata12.0 software (StataCorp, College Station, TX, USA). Fixed-effects model was used for low heterogeneity among studies (*I*^2^ < 50%) and random-effects model was deployed for high heterogeneity among studies (*I*^2^ ≥ 50%).

Among 748 potentially relevant published studies, 710 were excluded due to duplication (*n* = 126) or article types (*n* = 296) or because the title and abstract did not meet the inclusion criteria (*n* = 288). The retrospective study by Sakamoto et al. (10) enrolled 92 patients with cancers diagnosed prior to HF and thus was excluded. Thirty additional articles were excluded because they did not describe the outcome of interest. Finally, 8 cohort studies (4–7, 9, 11–13) with 384504 HF patients met all inclusion criteria and were included in the meta-analysis to assess the association between HF and cancer incidence. Characteristics of the 8 studies with HR adjusted for different comorbidities or risk factors are shown in Supplementary Table 1. Kwak et al. (4) reported both no lag and 2-year lag results and we only extracted 2-year lag analysis to reduce surveillance bias. Similarly, in Banke's study (12), data excluding all diagnosis of cancer within the 365 days after the diagnosis of HF was extracted.

**Figure 1:**
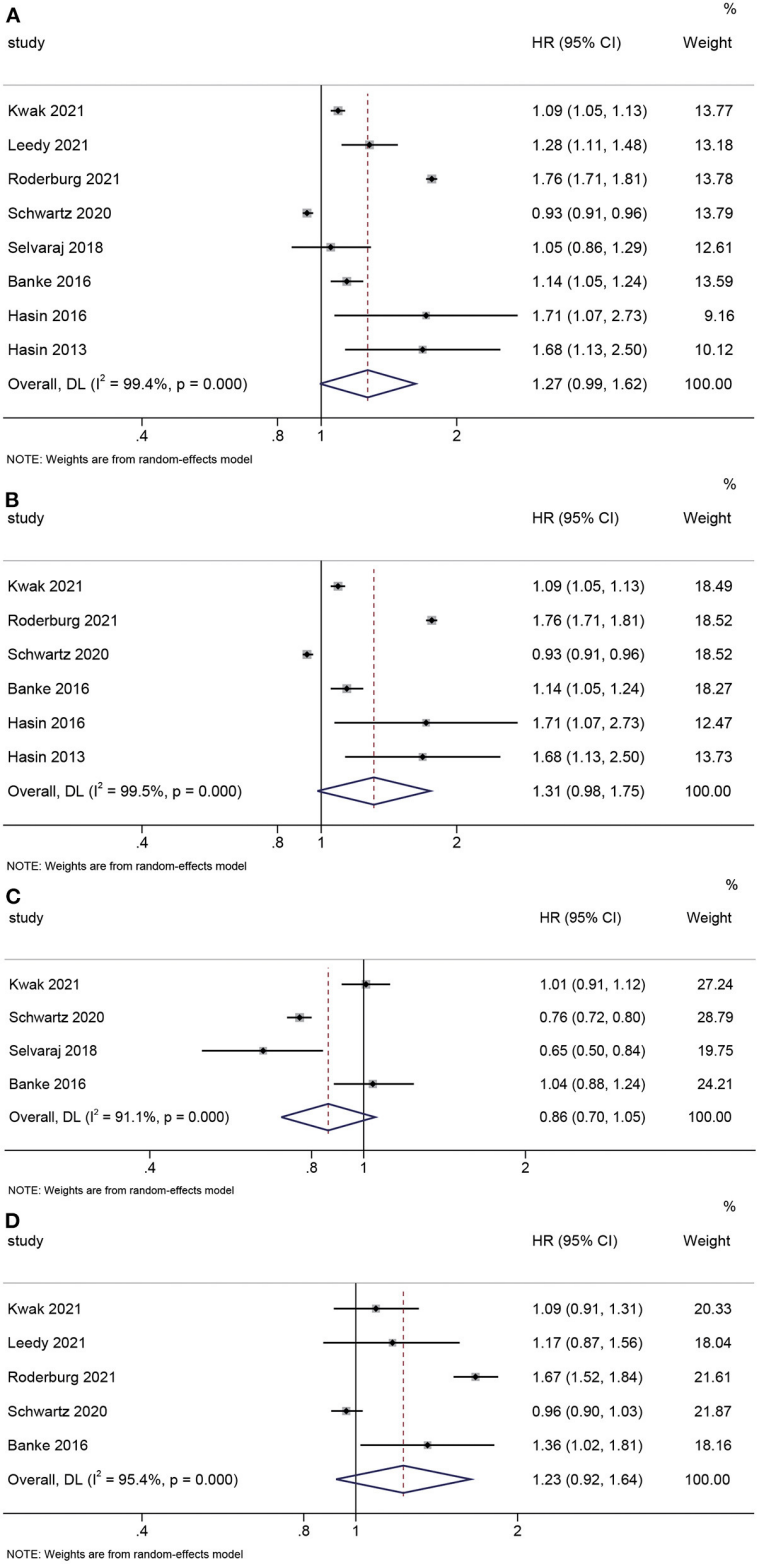
Forest plots. **(A)** Pooled hazard ratio for all types of cancer: HF vs. control. **(B)** Sensitivity analysis. **(C)** Pooled hazard ratio for prostate cancer: HF vs. control. **(D)** Pooled hazard ratio for breast cancer: HF vs. control.

The pooled HR of all types of cancer in HF patients from these 8 studies was 1.27 (95% CI: 0.99–1.62) without statistical significance (*P* = 0.056) (Figure 1A). Considering only male or female patients were enrolled in Leedy and Selvaraj's studies (5, 6), sensitivity analysis was done after excluding these 2 studies. The pooled HR changed little (1.31, 95% CI: 0.98–1.75) without statistical significance (*P* = 0.068) (Figure 1B). As to types of cancer, 4 studies provide data about male-specific prostate cancer and 5 studies present data about female-specific breast cancer. Our meta-analysis showed HF patients did not have an increased risk of prostate cancer (HR: 0.86, 95% CI: 0.70–1.05; *P* = 0.139) or breast cancer (HR: 1.23, 95% CI: 0.92–1.64; *P* = 0.166) (Figures 1C,D).

## Discussion

Our updated meta-analysis summarized all cohort studies presently available and demonstrated that there was no significantly increased risk of incident cancer among HF patients. In view of the high heterogeneity among published studies, bias may have existed. As presented in Kwak's study (4), an abrupt increase of new cancer diagnosis was observed in the first 2 years of HF diagnosis, but the difference in the cancer incidence became much smaller in the 2-year lag analysis, indicating that surveillance bias may partially account for the increased cancer incidence in HF patients. In addition, the higher cancer incidence among HF patients may be driven by comorbidities suggested in Schwartz's study (7). The increased cancer risk reduced substantially after adjustment for comorbidities and vanished for most cancer types after additional adjustment for baseline medication use (7). This showed that the highly controversial HF mediated cancer risk may be largely mediated by comorbidity risk and medications.

In conclusion, the available evidence still could not clearly identify HF as a new risk factor for incident cancer. Results should be interpreted with caution due to the high heterogeneity among studies. Further research is needed to elucidate the conflicting clinical data and uncover the underlying mechanisms.

## Author Contributions

XZ contributed to conceptualization, data collection, and writing. NL contributed to data collection, analysis, and editing. YZ contributed to analysis. JZ contributed to the conceptualization, illustration, and editing of the manuscript. All authors approved the submitted version.

## Funding

This work was supported by National Natural Science Foundation of China (82104588) and Pyramid Talent Program of Changzheng Hospital (YQ662) to JZ.

## Conflict of Interest

The authors declare that the research was conducted in the absence of any commercial or financial relationships that could be construed as a potential conflict of interest.

## Publisher's Note

All claims expressed in this article are solely those of the authors and do not necessarily represent those of their affiliated organizations, or those of the publisher, the editors and the reviewers. Any product that may be evaluated in this article, or claim that may be made by its manufacturer, is not guaranteed or endorsed by the publisher.
