# Supplementary material for: Is Heart Failure a New Risk Factor for Incident Cancer?
Source: Front Cardiovasc Med. 2022 Feb 7;9:828290. doi: 10.3389/fcvm.2022.828290 (PMC8858960; doi:10.3389/fcvm.2022.828290)
Supplement: Supplementary file 1 [file Data_Sheet_1.docx]

Supplementary Material

# Supplementary Table 1. Correlation between included variables.

|  | 1 | 2 | 3 | 4 | 5 | 6 | 7 | 8 | 9 |
| --- | --- | --- | --- | --- | --- | --- | --- | --- | --- |
| 1. Sex | 1 | 0.055^**^ | -0.001 | -0.029^*^ | -0.098^**^ | -0.120^**^ | 0.065^**^ | -0.128^**^ | 0.167^**^ |
| 2. Educational level |  | 1 | 0.118^**^ | 0.193^**^ | -0.075^**^ | -0.042^**^ | -0.015 | 0.058^**^ | -0.014 |
| 3. Title |  |  | 1 | 0.293^**^ | 0.492^**^ | -0.119^**^ | -0.101^**^ | 0.075^**^ | -0.031^*^ |
| 4. Monthly income |  |  |  | 1 | 0.213^**^ | -0.040^**^ | -0.046^**^ | 0.034^**^ | 0.038^**^ |
| 5. Years of service |  |  |  |  | 1 | 0.009 | -0.135^**^ | 0.135^**^ | -0.093^**^ |
| 6. Frequency of night shift |  |  |  |  |  | 1 | -0.137^**^ | 0.207^**^ | -0.149^**^ |
| 7. Perceived shortage of physicians |  |  |  |  |  |  | 1 | -0.245^**^ | 0.124^**^ |
| 8. Workplace verbal violence |  |  |  |  |  |  |  | 1 | -0.410^**^ |
| 9. Workplace physical violence |  |  |  |  |  |  |  |  | 1 |

* Correlation is significant at the 0.05 level (2-tailed). ** Correlation is significant at the 0.01 level (2-tailed).

# Supplementary Table 2. Characteristics of Effort-Reward Ratio (n = 10,457).

| Variable | All subjects  N (%) | Effort-Reward Ratio  Median (25th–75th percentiles) | *Z* | *P* |
| --- | --- | --- | --- | --- |
| **Total** | 10457(100) | 1.33(1.05-1.77) | - | - |
| **Sociodemographic characteristics** |  |  |  |  |
| Age | 31(28-35) ^&^ | - | - | - |
| Sex |  |  | -15.84 | <0.001^**^ |
| Male | 7632(72.98) | 1.38(1.09-1.83) |  |  |
| Female | 2825(27.02) | 1.21(0.98-1.57) |  |  |
| Educational level |  |  | 73.02 | <0.001^**^ |
| Associate degree or vocational diploma^#^ | 1684(16.10) | 1.26(1.00-1.63) |  |  |
| Bachelor degree | 7789(74.49) | 1.36(1.07-1.83) |  |  |
| Master degree or above | 984(9.41) | 1.26(1.00-1.69) |  |  |
| Marital status |  |  | -5.77 | <0.001^**^ |
| Unmarried/other | 1629(15.58) | 1.34(1.06-1.77) |  |  |
| Married | 8828(84.42) | 1.26(0.99-1.72) |  |  |
| **Work-related factors** |  |  |  |  |
| Title |  |  | 149.92 | <0.001^**^ |
| Junior or less | 4972(47.55) | 1.27(1.00-1.70) |  |  |
| Intermediate | 4112(39.32) | 1.40(1.11-1.83) |  |  |
| Senior | 1373(13.13) | 1.33(1.08-1.72) |  |  |
| Monthly income (YUAN) |  |  | 36.15 | <0.001^**^ |
| ≤4000 | 3862(36.93) | 1.36(1.04-1.83) |  |  |
| 4001~6000 | 3562(34.06) | 1.34(1.06-1.77) |  |  |
| ≥6001 | 3033(29.00) | 1.29(1.03-1.67) |  |  |
| Years of service |  |  | 268.62 | <0.001^**^ |
| <1 | 1448(13.85) | 1.17(0.94-1.52) |  |  |
| 1-5 | 3965(37.92) | 1.30(1.02-1.76) |  |  |
| 6-10 | 2458(23.51) | 1.40(1.10-1.90) |  |  |
| ≥11 | 2586(24.73) | 1.41(1.11-1.83) |  |  |
| Frequency of night shift (per month) |  |  | 278.21 | <0.001^**^ |
| 0~5 | 2033(19.44) | 1.19(0.97-1.55) |  |  |
| 6~10 | 5633(53.87) | 1.33(1.05-1.76) |  |  |
| ≥11 | 2791(26.69) | 1.45(1.13-1.99) |  |  |
| Perceived shortage of physicians |  |  | 970.47 | <0.001^**^ |
| Not meet demands | 7667(73.32) | 1.42(1.13-1.90) |  |  |
| General | 1856(17.75) | 1.14(0.94-1.44) |  |  |
| Meet demands | 934(8.93) | 1.01(0.82-1.34) |  |  |
| Workplace verbal violence (times) |  |  | 1311.97 | <0.001^**^ |
| 0 | 1902(18.19) | 1.05(0.88-1.38) |  |  |
| 1~3 | 4130(39.50) | 1.26(1.02-1.62) |  |  |
| ≥4 | 4425(42.32) | 1.56(1.22-2.04) |  |  |
| Workplace physical violence |  |  | 28.28 | <0.001^**^ |
| Yes | 2889(27.63) | 1.61(1.25-2.17) |  |  |
| No | 7568(72.37) | 1.26(1.00-1.62) |  |  |

^*^ *P-value* < 0.05, ^**^ *P-value* < 0.001

^&^ The age was reported using median (25th–75th percentiles).

^#^ An associate degree refers to students graduating from senior middle school (grade year 10 to year 12) receiving 3 years of education in college, or students graduating from junior middle school (grade year 7 to year 9) receiving 5 years of education in college.

A vocational diploma refers to students graduating from senior middle school receiving 2 years of education in vocational schools or students graduating from junior middle school receiving 3 years of education in vocational schools.
